# The intact parasympathetic nerve promotes submandibular gland regeneration through ductal cell proliferation

**DOI:** 10.1111/cpr.13078

**Published:** 2021-06-07

**Authors:** Xue Wang, Zhilin Li, Qi Shao, Chunmei Zhang, Jinsong Wang, Zhengxue Han, Songlin Wang, Lizheng Qin

**Affiliations:** ^1^ Salivary Gland Disease Center and Beijing Key Laboratory of Tooth Regeneration and Function Reconstruction Capital Medical University School of Stomatology Beijing China; ^2^ Department of Oral and Maxillofacial and Head and Neck Oncology Capital Medical University School of Stomatology Beijing China; ^3^ Department of Biochemistry and Molecular Biology Capital Medical University School of Basic Medicine Beijing China; ^4^ Immunology Research Center for Oral and Systemic Health Beijing Friendship Hospital Capital Medical University Beijing China; ^5^ Laboratory for Oral and General Health Integration and Translation Beijing Tiantan Hospital Capital Medical University Beijing China

**Keywords:** ductal cell proliferation, neural cell adhesion molecule (NCAM), parasympathetic innervation, polysialic acid (PSA), submandibular gland regeneration

## Abstract

**Objectives:**

Salivary gland regeneration is closely related to the parasympathetic nerve; however, the mechanism behind this relationship is still unclear. The aim of this study was to evaluate the relationship between the parasympathetic nerve and morphological differences during salivary gland regeneration.

**Materials and Methods:**

We used a duct ligation/deligation‐induced submandibular gland regeneration model of Sprague‐Dawley (SD) rats. The regenerated submandibular gland with or without chorda lingual (CL) innervation was detected by haematoxylin–eosin staining, real‐time PCR (RT‐PCR), immunohistochemistry and Western blotting. We counted the number of Ki67‐positive cells to reveal the proliferation process that occurs during gland regeneration. Finally, we examined the expression of the following markers: aquaporin 5, cytokeratin 7, neural cell adhesion molecule (NCAM) and polysialyltransferases.

**Results:**

Intact parasympathetic innervation promoted submandibular gland regeneration. The process of gland regeneration was significantly repressed by cutting off the CL nerve. During gland regeneration, Ki67‐positive cells were mainly found in the ductal structures. Moreover, the expression of NCAM and polysialyltransferases‐1 (PST) expression in the innervation group was significantly increased during early regeneration and decreased in the late stages. In the denervated submandibular glands, the expression of NCAM decreased during regeneration.

**Conclusions:**

Our findings revealed that the regeneration of submandibular glands with intact parasympathetic innervation was associated with duct cell proliferation and the increased expression of PST and NCAM.

## Graphical abstract

Intact parasympathetic nerve innervation promoted submandibular gland regeneration. The process of gland regeneration was significantly repressed by cutting off the parasympathetic nerve. During regeneration, the proliferating cells were mainly found in the ductal structures with parasympathetic innervation. On the contrary, almost no proliferative ductal cells were found in the denervation group. This differential pattern of cell proliferation is most associated with the increased expression of PST and NCAM.

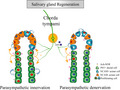

## 1 INTRODUCTION

Hyposalivation is caused by multiple diseases, such as radiation‐induced xerostomia, Sjӧgren's syndrome and other salivary cancers.^1^, ^2^, ^3^ The current treatments for hyposalivation, such as saliva secretion stimulators or artificial saliva, can only alleviate the symptoms temporarily. Severe xerostomia still lacks effective treatments. Therefore, it is vital to investigate how to restore dysfunctional salivary glands.

Regeneration therapies have the potential to recover salivary gland function. Many researchers have investigated the mechanisms responsible for the development of salivary gland.^4^, ^5^ The parasympathetic nerve is involved in the initial stage of gland organogenesis and accompanies the entire process of gland development.^4^, ^6^ Parasympathetic nerves release acetylcholine, which activates muscarinic M3 receptors, evoking a normal flow of saliva.^7^ The chorda lingual (CL) nerve contains parasympathetic fibres, which run parallel with main secretory duct at the hilum into the submandibular and sublingual glands.^8^, ^9^ It has been reported that the parasympathetic nerve maintains a reservoir of progenitor cells for salivary organogenesis.^6^ During submandibular gland (SMG) organogenesis, the parasympathetic coordinates multiple steps in tubulogenesis. Studies have found that the ductal tubulogenesis of SMG is impaired in the absence of the parasympathetic nerve.^10^ Furthermore, parasympathetic neuronal function promotes epithelial organ regeneration after radiation damage.^6^ However, the role of the parasympathetic nerve in gland regeneration is not fully understood.

Neural cell adhesion molecule (NCAM) is a surface glycoprotein, and it is mainly expressed in the developing and regenerating nervous system, where it participates in myelination and promotes axon growth.^11^, ^12^ The expression of NCAM decreases significantly after cutting off the peripheral nerve and plays an important role in subsequent nerve regeneration.^13^ Consistently, axon loss and abnormal axon projection in sensory nerves were found in NCAM knockout mice.^14^ Importantly, NCAM requires post‐translational modifications to better perform its biological functions, such as combining with polysialic acid (PSA) to form polysialylated‐NCAM (PSA‐NCAM).^15^ The combination of PSA and NCAM is an enzymatic reaction catalysed by two polysialyltransferases (polySTs), ST8siaII (STX) and ST8siaIV (PST).^16^, ^17^ PSA with a large number of negative charges can reduce the adhesion of NCAM, promote cell migration and play an important role in the formation and reconstruction of the nervous system.^15^, ^18^ Interestingly, in the field of liver regeneration, it has been found that the complex of PSA‐NCAM was activated after liver damage, thereby modulating the migration, differentiation and maturation of liver progenitor cells with differentiation potential to repair the damaged liver.^19^ Moreover, the expression of PSA‐NCAM is reduced in the brain of patients with Alzheimer and Parkinson diseases.^20^ It has been reported that PSA‐NCAM can be used as a predictor of neural cell repair after unilateral recurrent laryngeal nerve injury.^21^ Fukuda et al reported that NCAM was generally expressed in human submandibular salivary gland tumour cell line (HSG).^22^ However, the roles of PSA‐NCAM in mediating the regeneration of salivary gland after cutting off the parasympathetic nerve are unknown.

In the current study, we used an SMG duct ligation/deligation‐induced regeneration rat model to identify the role of parasympathetic innervation. We further detected differences in regenerating gland proliferation in the innervation and denervation groups. Finally, we confirmed PSA‐NCAM as a potential indicator of salivary gland regeneration after parasympathetic nerve injury.

## 2 MATERIALS AND METHODS

### 2.1 Animals

Forty‐eight 6 weeks‐old Sprague‐Dawley male rats (around 150‐170 g) were purchased from SPF Biotechnology co., Ltd. All experiments were conducted in accordance with the guide for the care and use of animals by the National Institute of Health. All animals were maintained under standard conditions and had free access to water and food. The experimental part was approved by the Animal Care and Use Committee of Beijing Stomatological Hospital affiliated to Capital Medical University (Ethical code: No. 201702‐001).

### 2.2 Experimental procedures

All experimental procedures were performed under aseptic conditions. The left submandibular gland (LSM) was chosen for the experimental side, and the contralateral right gland (RSM) was served as an untreated control. The rats were divided into three groups randomly: non‐treated, innervation and denervation (n = 16 per group). Animals in the non‐treated group were performed sham surgery. Animals were anaesthetized by intraperitoneal injection of 50 mg/kg of sodium pentobarbital (Sigma). The ligation of SMG main excretory duct and the dissection of parasympathetic nerve were performed as previously described.^9^, ^23^ Under the vertical skin incision, the duct was ligated using 4‐0 vicryl surgical sutures (Ethicon), and the CL was cut off in the denervation group. Animals in the non‐treated group only had incision and were not treated with ducts and CL nerves. The incision was sutured closely with 2‐0/T black silk sutures (Ethicon). Then, the ligation was removed after one week. At days 0, 7, 14 and 28 after duct deligation, four animals in each group were sacrificed with an anaesthetic overdose at each time point, and the glands of both sides were harvested and weighted. The glands of LSM from three groups were quickly divided into three parts and then processed for histological examination, Western blot and real‐time PCR (RT‐PCR) analysis as described below.

### 2.3 Histological analyses

Tissues were fixed with 4% paraformaldehyde, dehydrated with gradient ethanol and then embedded in paraffin. Sections (5 μm thickness) were stained with haematoxylin and eosin (H&E) staining and examined using microscope (OLYMPUS).

### 2.4 Immunofluorescence (IF) and histochemistry (IHC) staining

The sections were deparaffinized with xylene and rehydrated with a series of ethanol solutions in phosphate‐buffered saline (PBS). After that, the tissue sections were processed with antigen retrieval by boiling the slides in sodium citrate buffer (10 mmol/L, pH 6.0) for 20 minutes. Then, the sections were immersed in 10% H_2_O_2_/methanol for 10 minutes to block the endogenous peroxidases activity, followed by PBS with 5% BSA and 0.2% Triton X‐100 at room temperature for 1 hour. The sections were incubated with primary antibodies overnight at 4°C. After washing with PBS for 10 minutes three times, sections were incubated with secondary antibodies (Alexa Fluor or horseradish peroxidase‐conjugated series) at room temperature for 2 hours. IF images were taken using a Leica confocal microscope. IHC were developed with 3,3'‐diaminobenzidine substrate and counterstained with haematoxylin; the images were captured under a microscope.

The primary antibodies used were as follows: rabbit polyclonal anti‐aquaporin 5 (1:2000 dilution, ab78486, Abcam), rabbit polyclonal anti‐ki67 (1:200 dilution, ab16667, Abcam), mouse monoclonal anti‐cytokeratin 7 (1:200 dilution, MA1‐06315, Thermo Fisher Scientific), rabbit polyclonal anti‐PST (1:100 dilution, A6754, Abclonal), rabbit monoclonal anti‐NCAM1 (1:500 dilution, ab220360, Abcam) and mouse monoclonal anti‐PCNA (1:1000 dilution, 2586S, Cell Signaling Technology).

The secondary antibodies used were as follows: mouse anti‐rabbit IgG‐HRP (1:200 dilution, SC‐2357, Santa Cruz Biotechnology), donkey polyclonal anti‐rabbit IgG (H + L) Alexa Fluor 594 (1:1000 dilution, A‐21207; Thermo Fisher Scientific), donkey polyclonal anti‐mouse IgG (H + L) and Alexa Fluor 488 (1:1000 dilution, A‐21202; Thermo Fisher Scientific).

The negative controls were as follows: PBS instead of the primary antibody was used as the secondary antibody only control. Mouse monoclonal anti‐IgG (1:200 dilution, SC‐51643, Santa Cruz Biotechnology) instead of the primary antibody was used as negative control.

### 2.5 RNA isolation, RT‐PCR and Real‐Time RT‐PCR

Total RNA was extracted from glands using TRIzol reagent according to the manufacturer's instructions (Invitrogen). Next, we synthesized cDNA with a Prime Script RT Reagent Kit (Takara). Gene transcripts were quantified via real‐time PCR performed with SYBR Green PCR Kit (Qiagen). The sequences for the primers used were listed in Table S1. The relative gene expression was normalized to *β‐actin* levels and determined by the 2^(−△△Ct)^ method.

### 2.6 Western blot

The collected glands were lysed in RIPA Lysis and Extraction Buffer (Thermo Fisher Scientific). Individual gland lysates were loaded and separated by sodium dodecyl sulphate–polyacrylamide gel electrophoresis (SDS‐PAGE) and then transferred to an Immobilon‐P polyvinylidene difluoride membrane (Millipore). After blocking with 5% milk in PBS, the membrane was incubated with primary antibodies rabbit monoclonal anti‐NCAM1 (1:1000 dilution, ab220360, Abcam) and rabbit polyclonal anti‐muscarinic acetylcholine receptor M3 (1:1000 dilution, bs‐1289R, Bioss) at 4°C overnight. After incubation with secondary antibodies, goat anti‐rabbit IgG‐HRP (1:2000 dilution, SC‐2004; Santa Cruz Biotechnology) at room temperature, membranes were used western ECL substrate (Bio‐Rad), followed by exposure of the membranes to film and digital imaging.

### 2.7 Statistical analysis

All experiments were randomized into groups by block randomization. Data collection and analysis were performed blindly. No samples and animals were excluded from analysis. Comparisons between two groups were performed using unpaired two‐tailed Student's *t* tests; one‐way analysis of variance (ANOVA) was used for comparisons between more than two groups. Data were expressed as the means ± standard deviation. Statistical analyses and graphical generation of data were done with GraphPad Prism 8.0. Differences were considered significant at the value of *P* < .05.

We used Image‐Pro Plus version 6.0 software to analyse the sum Integral optical density (IOD) of AQP5, NCAM and PST protein in four glands from four animals in each group and five fields in each gland. Ki67 and CK7 proteins are markers of cell proliferation and salivary ductal cells, respectively. We calculated the number of Ki67 + cells per field at × 400 magnification using ImageJ software in four glands from four animals in each group and five fields in each gland.

## 3 RESULTS

### 3.1 Complete parasympathetic innervation facilitates gland regeneration

Initially, we selected a rat model of submandibular gland regeneration, in which the main excretory duct was ligated for one week and the ligation was subsequently removed. Then, the bilateral submandibular glands were collected for analysis at 0, 7, 14 and 28 days after duct deligation (four animals in each group at each time point). We found that the glands were atrophic, and their weight decreased significantly at 0 days. Through continuous observation, we found that the weights and morphology of these glands almost recovered to the level of the contralateral glands at the 28th days after deligation.

**FIGURE 1:**
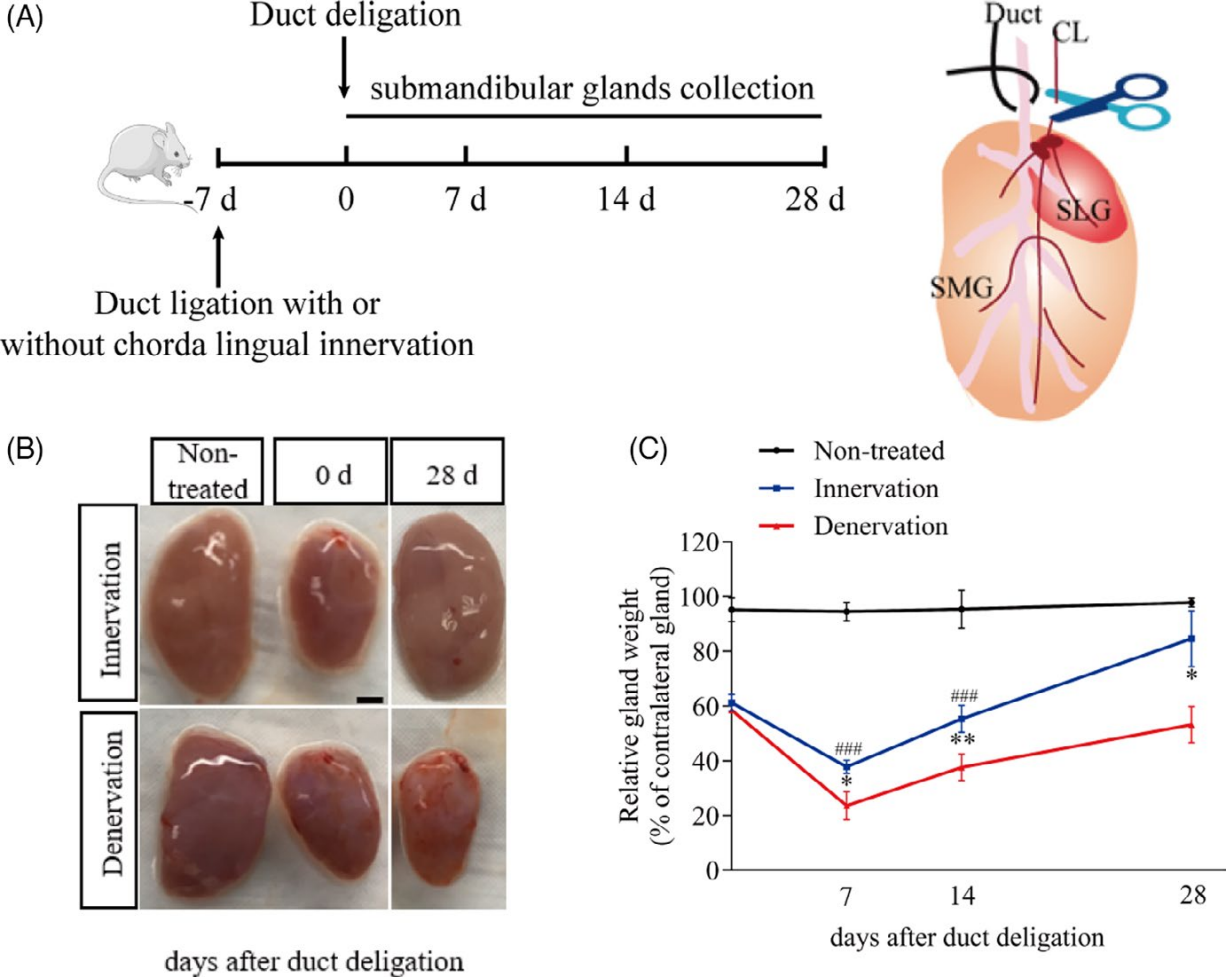
Complete parasympathetic innervation facilitates gland regeneration. (A) The specific process of this experiment. CL, chorda lingual. SMG, submandibular gland. SLG, sublingual gland. (B) The pictures of submandibular glands (SMG) in different groups after duct deligation. The non‐treated group were performed sham surgery and serves as a control (scale bar = 1mm). (C) The ratio of left gland weight to right gland in each group at different time points (0, 7, 14, 28 after duct deligation) during regeneration. The weight of innervated glands was significant recovered compared with the denervated (*indicates significance between the group innervation and denervation. # indicates significance between the non‐treated group and innervation group). *P < .05, ** and *P* < .01, ### *P* < .001). Data were shown as mean ± standard deviation

Next, we used established models to investigate whether parasympathetic nerves play a role in gland regeneration. Forty‐eight rats were used in this study (three groups, n = 16 per group). The specific experimental procedure is shown in Figure 1A. Muscarinic acetylcholine receptor M3 (CHRM3) and nicotinic acetylcholine receptor β1 (CHRNB1) were used as markers of the parasympathetic nerve.^24^ Brain‐derived neurotrophic factor (BDNF) is known as a potential survival factor for neurons.^25^ We found that *Chrm3*, *Chrnb1* and *Bdnf* expression levels were significantly decreased compared with non‐treated glands after cutting off the parasympathetic nerve (Figure S1A). Moreover, the protein levels of NCAM and CHRM3 were markedly reduced after CL denervation (Figure S1B). Compared with the untreated glands in the non‐treated group, the glands in the other two groups were atrophic and their texture was hard at day 0; however, it was recovered at day 28 (Figure 1B). Standardized with the contralateral glands, the weight of the innervation was decreased by approximately 40% at day 0 and 63% at day 7, and the weight gradually increased to 55% at day 14 (*P* < .001). We observed that on day 28 after deligation, the gland morphology recovered and the weight returned to 84% of the non‐treated glands (*P* > .05) (Figure 1C). However, the weight of denervated glands was decreased by approximately 42% at day 0 and 77% at day 7. The weight of glands in the denervation group also increased to 38% of that in the untreated group at day 14 and 53% at day 28 (*P* < .05). However, the glands were still abnormal and hard (Figure 1B). These data indicate that intact parasympathetic innervation significantly promotes gland regeneration.

### 3.2 Intact parasympathetic innervation promotes the recovery of gland morphology and function

**FIGURE 2:**
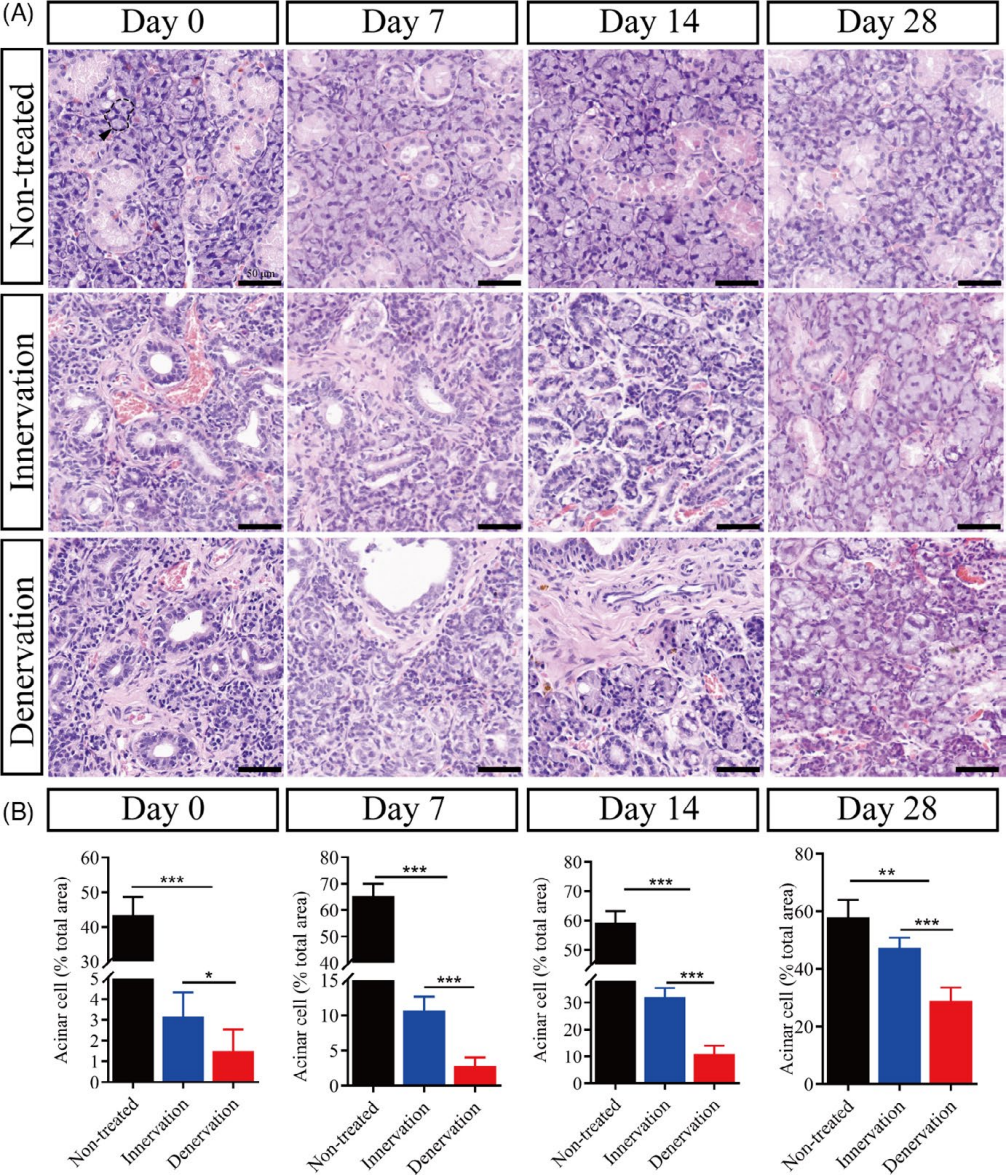
Intact parasympathetic innervation promotes the recovery of gland morphology. (A) Haematoxylin and eosin (H&E) staining of submandibular glands in the non‐treated, innervation and denervation group at days 0, 7, 14 and 28 after duct deligation. Scale bar = 50 μm. Arrowhead indicates acinus. (B) The number of acinar cells in the field between different groups at different times after duct deligation. *** indicates significance at *P* < .001, ** *P* < .01, * *P* < .05. Data were shown as mean ± standard deviation

To confirm the role of the parasympathetic nerve, we observed morphological changes during gland regeneration. Histological analysis with haematoxylin and eosin (H&E) staining showed intensive secretory acinus in the non‐treated group. Compared to the untreated glands, the glands in the two operated groups were characterized by a significant reduction in acinar cell number, acinar atrophy, duct dilatation and inflammatory cell infiltration at day 0 after duct deligation (Figure 2). In the denervation group, severe fibrosis was found in most of the gland regions. Seven days after deligation, a few newly formed acinar cells and some duct‐like structures were observed in the innervation group, and the number of newly formed acinar cells increased in the following days. Twenty‐eight days after deligation, only a small number of atrophic acinar cells were found in the innervation group, which showed no significant difference in morphology compared with the non‐treated group (Figure 2B). On the contrary, the newly formed acinar cells were scattered around the glands at day 7 in the denervation group. Although the number of newly formed acinar cells increased slowly at days 14 and 28, there was more fibrotic tissue around the duct and more infiltration of mononuclear cells in the tissue than in the innervation group (Figure 2B).

**FIGURE 3:**
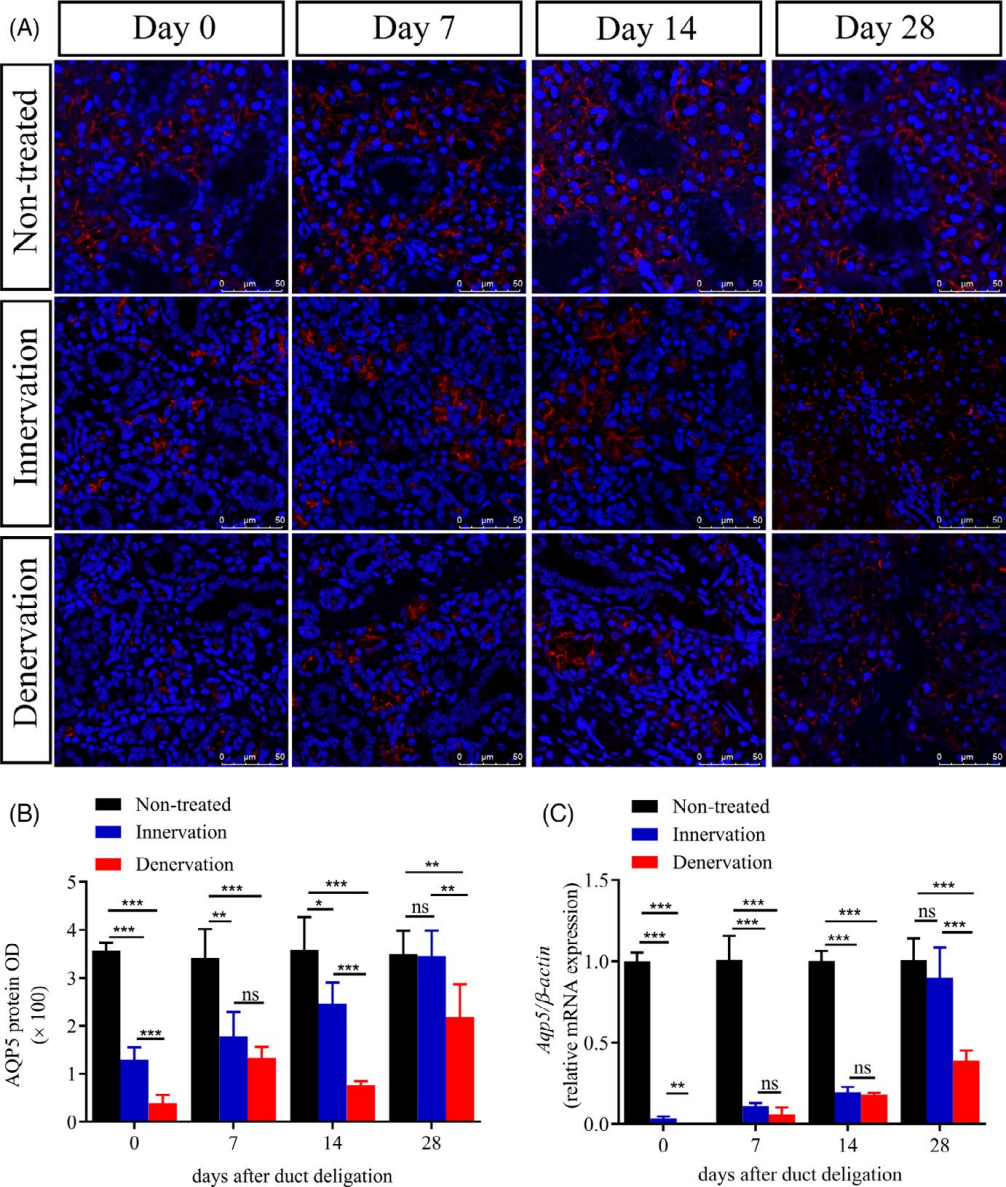
Intact parasympathetic innervation promotes the recovery of gland function. (A) Immunofluorescence (IF) of aquaporin 5 protein (AQP5) and DAPI (blue) in the gland of non‐treated, innervation and denervation groups at 0, 7, 14 and 28 days after deligation. Scale bar = 50 μm. (B) Relative IF expression levels of AQP5 at different times. (C) AQP5 mRNA levels in different groups. *** indicates significance at *P* < .001, ** *P* < .01, * *P* < .05, ns for no significant. Data were shown as mean ± standard deviation

Aquaporin 5 (AQP5) is an important protein for evaluating the normal physiological function of the salivary gland.^26^ In the non‐treated group, immunofluorescence (IF) staining showed that AQP5 was strongly expressed in acinar cells (Figure 3, Figure S3). At day 0, whether the CL was intact or not, it was observed that the protein expression of AQP5 was significantly decreased (*P* < .001). Interestingly, the protein expression of AQP5 was significantly reduced in the denervation group (*P* < .001). At day 7, the protein expression of AQP5 was increased slightly in the two experimental groups compared with that at day 0. It was found that the proportion of shrunken acinar cells was also high in the denervation group. Compared with day 7, we observed that the expression of AQP5 was still increased in the innervation group, but was decreased in the denervation group at day 14. In the denervation group, AQP5 protein level was decreased. At day 28, the level of AQP5 protein in the innervation group recovered to 98% (*P* > .05) of the non‐treated group, while that in the denervation group only recovered to 62% (*P* < .01) (Figure 3A,B). Then, we checked the mRNA expression of *Aqp5*, which was normalized to that of non‐treated glands (Figure 3C). The restoration of AQP5 expression might suggest that the physiological state of the gland was recovered to some extent.

### 3.3 Parasympathetic innervation increases ductal epithelial cell proliferation

**FIGURE 4:**
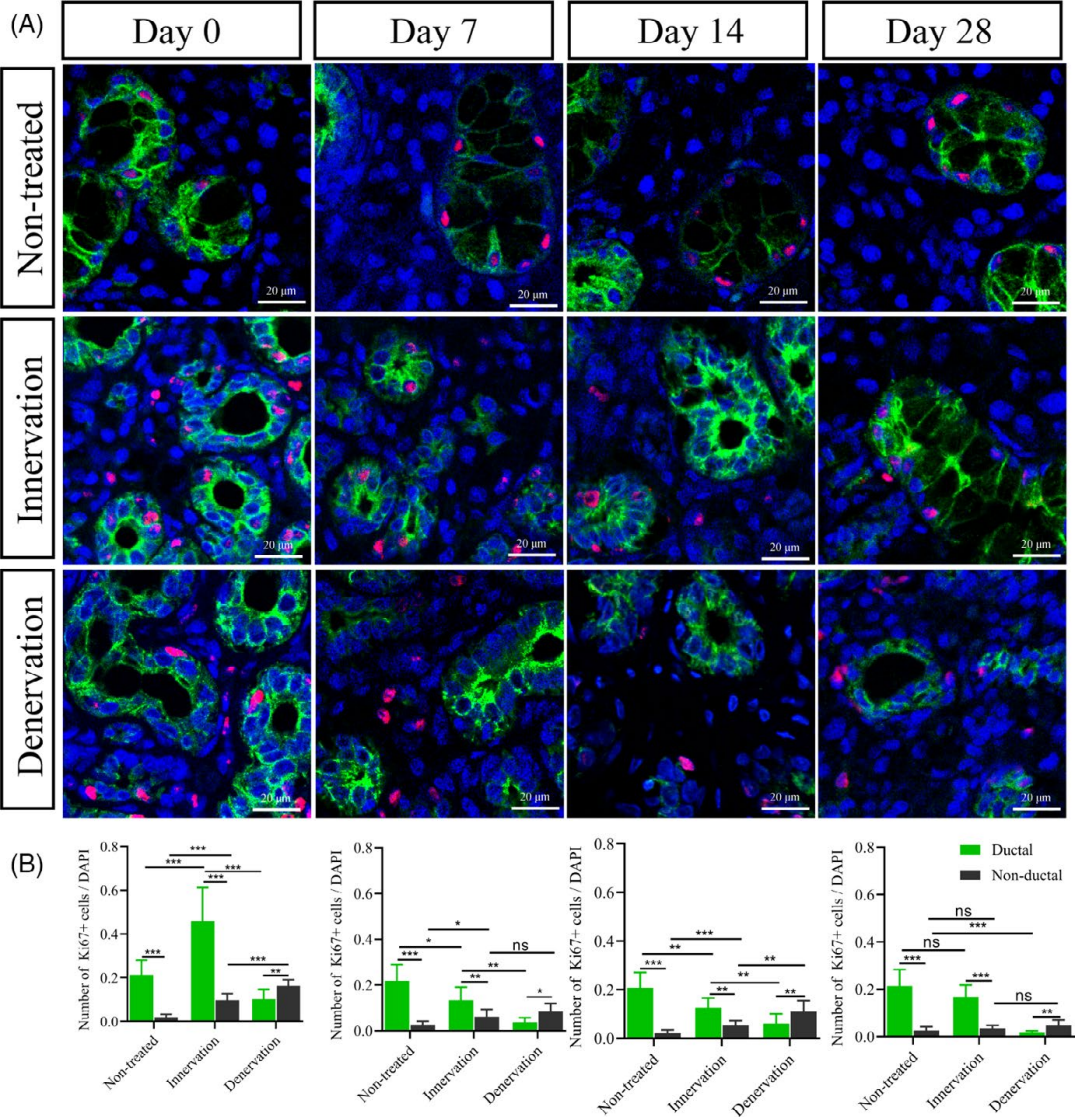
Parasympathetic innervation increases ductal epithelial cell proliferation. (A) Immunofluorescence staining of cytokeratin 7 (green), Ki67 (red) and DAPI (blue) in the submandibular glands during gland regeneration in different groups. Scale bar =20 μm. (B) The number of Ki67+ in ductal and non‐ductal cells was different. The relative expression of ki67+ was maintained a stable state at days 14 and 28. *** indicates significance at *P* < .001, ** *P* < .01, * *P* < .05, ns for no significant. Data were shown as mean ± standard deviation

To determine how these atrophic glands recover their function, we detected the expression of Ki67, proliferating cell nuclear antigen (PCNA) and cytokeratin 7 (CK7) at different times. Ki67 and PCNA proteins are markers of cell proliferation, and CK7 is the marker of salivary ductal cells, respectively.^27^, ^28^ In the non‐treated group, CK7 was localized to the intercalated duct, striated duct, granular and excretory duct cells. Ki67 was mainly expressed in these CK7‐positive (CK7+) ductal cells, and a small number of Ki67‐positive cells (Ki67+) were located in non‐ductal tissues. We found that the number of Ki67+ cells increased significantly between the two operated groups (Figure 4, Figure S2,S3). However, the majority of Ki67+ cells in the innervation group were located in the ductal tissue (CK7+Ki67+) and the remaining cells were in the non‐ductal tissue (CK7‐Ki67+) in the early stages of gland regeneration (days 0 and 7). Fourteen days after duct deligation, the salivary glands were in a stable state. The number of Ki67+ cells decreased gradually, and the ratio of CK7+Ki67+ to CK7‐Ki67+ was recovered to the level of non‐treated glands at days 14 and 28. In the denervation glands, Ki67+ cells were almost all expressed in non‐ductal tissues, and only a small number of Ki67+ cells were expressed in the ducts during regeneration. The number of Ki67+ cells in the denervation group decreased on day 7 and maintained a stable state of cell proliferation at days 14 and 28. Similar expression of PCNA was obtained during gland regeneration (Figure S2). Thus, different cell proliferation patterns were observed in the operated groups during the gland regeneration (Figure 4). Taken together, these results indicate that the intact parasympathetic innervation contributes to salivary gland regeneration through the ductal cell proliferation.

### 3.4 Parasympathetic nerve promotes ductal proliferation associated with PST and NCAM

**FIGURE 5:**
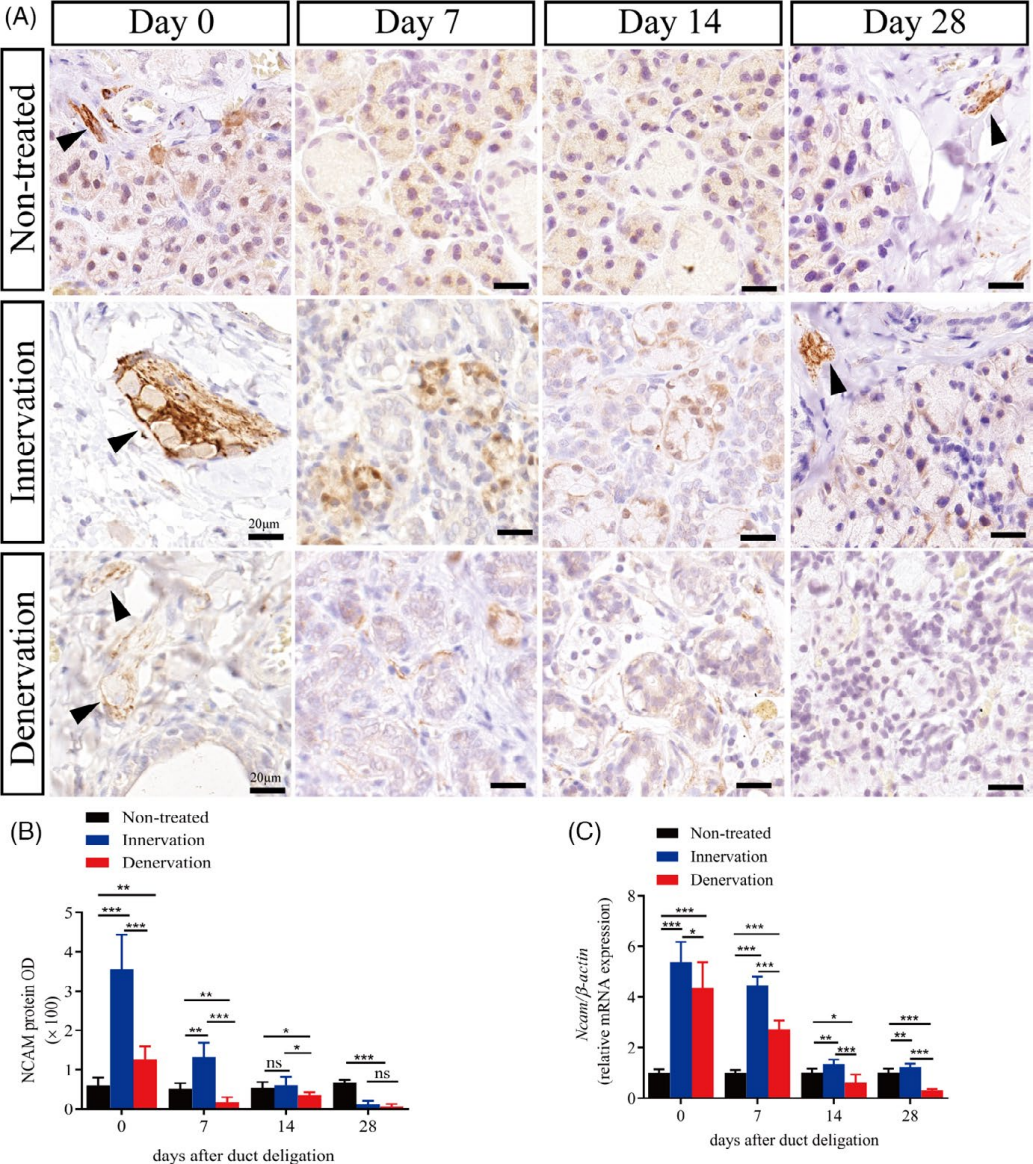
Parasympathetic nerve promotes NCAM expression in the regenerating glands. (A) Immunohistochemistry (IHC) of neural cell adhesion molecule (NCAM) in the submandibular gland of non‐treated, innervation and denervation groups. Scale bar = 20 μm. Arrowhead indicates nerve fibres. (B) Relative IHC expression of NCAM in the different groups at days 0, 7, 14, 28 after duct deligation. (C) NCAM mRNA levels in the different groups. *** indicates significance at *P* < .001, **P < .01, *P < .05, ns for no significant. Data were shown as mean ± standard deviation

PSA‐NCAM is a predictor of neural cell repair after peripheral nerve injury and modulates liver regeneration.^19^, ^21^ Immunohistochemistry was used to investigate the expression of NCAM and polySTs during gland regeneration. Under normal conditions, NCAM protein is expressed at a very low and stable level. NCAM was observed in the acinar cells and nerve fibres around the ducts (Figure 5, Figure S4). In the innervation group, NCAM expression was increased significantly (days 0 and 7) and decreased gradually to a stable state at days 14 and 28 compared with non‐treated glands at both the mRNA and protein levels (Figure 5C). However, the number of NCAM‐positive cells in the denervation group was only 2.1 times that of the non‐treated group at day 0 (*P* < .01). The expression of NCAM decreased significantly in the following days. In the denervation group, the mRNA expression of *Ncam* was decreased obviously at day 28. (Figure 5C).

**FIGURE 6:**
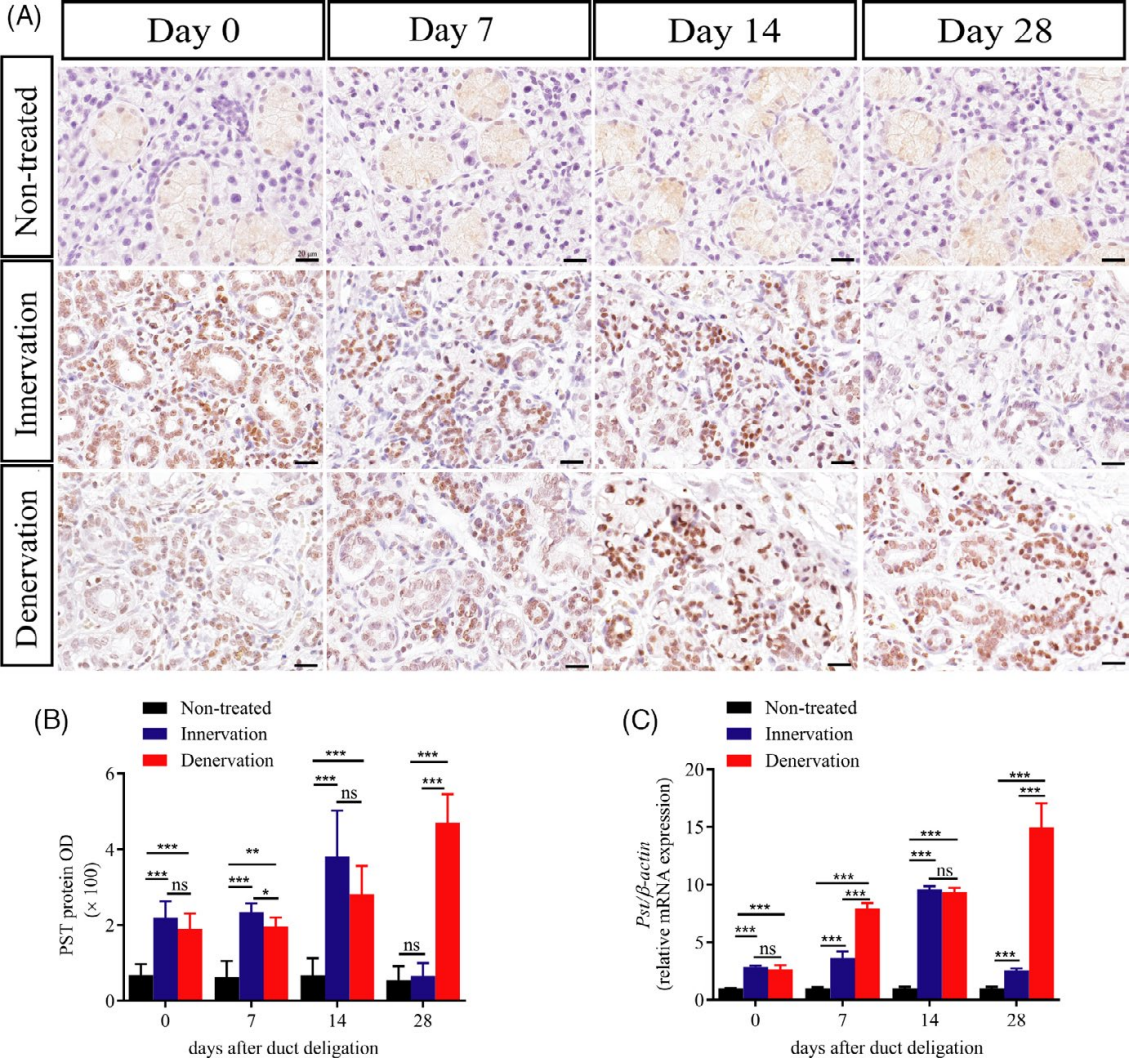
Parasympathetic nerve promotes PST expression in the regenerating glands. (A) Immunohistochemistry (IHC) of polysialyltransferases IV (PST) in the different groups. Scale bar = 20μm. (B) Relative IHC expression levels of PST at different times. (C) PST mRNA levels in glands in the different groups. *** indicates significance at *P* < .001, **P < .01, *P < .05, ns for no significant. Data were shown as mean ± standard deviation

We next detected the expression of two enzymes polyST enzymes in the salivary glands, STX and PST, which produced PSA. PSA is a unique post‐translational modifier of NCAM and regulates cell adhesion and cell migration.^15^ We found that the mRNA expression of *Pst* was increased in the innervation group at days 0, 7 and 14 after deligation, but decreased at day 28. However, the mRNA expression of *Pst* in the denervation group continued to increase (Figure 6C). Intriguingly, we did not find any changes in STX in any of the samples (data not shown). Subsequently, we investigated the changes in PST by IHC. In the untreated group, we found that PST was positively expressed in the cytoplasm of intercalated ducts, striated ducts, granular ducts, intralobular ducts and interlobular ducts (Figure 6, Figure S4). Using the non‐treated group as the baseline, we found that the number of PST‐positive cells (PST+) in the innervation group gradually increased and declined to normal levels at day 28. In the denervation group, PST+cells gradually increased, which was 8.48 times that of the untreated group at day 28 (Figure 6). Moreover, the location of PST was transferred from the cytoplasm to the nucleus. These data indicated that the regeneration of submandibular glands with intact parasympathetic innervation was associated with the increased expression of PST and NCAM.

## 4 DISCUSSION

Saliva secretion is mainly regulated by parasympathetic innervation.^4^, ^5^ In this study, we established a rat model of submandibular gland regeneration using unilateral CL injury and investigated the role of parasympathetic nerves during gland regeneration. We demonstrated that the intact parasympathetic nerve promotes salivary gland regeneration. The pattern of proliferating cells during regeneration was different in the innervation and denervation groups. This differential pattern of cell proliferation in the gland regeneration is associated with the increased expression of PST and NCAM.

Salivary glands have a remarkable ability to recover their function by regenerating the secretory tissue after duct ligation is reversed.^23^, ^29^ Interestingly, without parasympathetic nerve supply, ductal tubulogenesis was aberrant during the embryonic period, which results in atrophy of the adult submandibular gland.^10^, ^30^ First, we cut off the CL nerve and detected the mRNA expression levels of parasympathetic neural markers *Chrm3*, *Chrnb1*, and *Bdnf* were reduced. The protein levels of NCAM and CHRM3 were markedly reduced after denervation. Then, we used the rat model of duct ligation/deligation with or without CL innervation to detect the role of parasympathetic nerve in the submandibular gland regeneration. After one week of ligation, we observed the disappearance of numerous acinar cells, dilatation of ducts, infiltration of inflammatory cells and extensive fibrosis in the gland, as previously reported.^9^, ^29^ At day 7 after deligation, a small number of newly formed acinar cells were observed near the duct. The number of newly formed acinar cells in the innervation group was higher than that in the denervation group. The presence of numerous newly formed acinar cells and the increased number of AQP5+ cells in the restored glands may indicated that the gland gradually restored its secretory function. The difference between the innervation and denervation groups illustrates the importance of intact parasympathetic innervation for gland regeneration.

Previous studies suggested that the regeneration of salivary glands involves both self‐duplication of the remaining acinar cells and differentiation of new acinar cells.^29^, ^31^, ^32^ Acinar cells can be regenerated through either acinar or duct cell after severe damage, and cellular plasticity in regenerating glands was revealed by lineage tracing in vivo.^33^ The parasympathetic nerve improves embryonic salivary gland branching morphogenesis and regulates ductal tubulogenesis during organogenesis.^6^, ^10^ In this study, we used CK7 as a salivary duct marker to detect differences in regenerating gland proliferation in the innervation and denervation groups. Under normal conditions, the proliferative cells were mainly located in the ducts, and the rest were non‐ductal cells. We found that in both the operated groups, the cell proliferation patterns of ductal and non‐ductal cells were different. Regeneration is a healing process based on cell proliferation. The number of Ki67+ cells increased significantly in the early stages of regeneration and decreased gradually to a stable level in the late stage. Our data indicated that the number of Ki67+ cells in the ductal tissue was much greater than that in the non‐ductal tissues in the innervation group. On the contrary, almost no proliferative ductal cells were found in the denervation group. The different proliferation patterns in innervation and denervation demonstrate that the parasympathetic nerve facilitates gland regeneration through ductal cell proliferation. During the salivary gland regeneration, the proliferation of ductal cells may be concerned with acinar cell regeneration.

It has been reported that the expression of NCAM decreases after peripheral nerve injury, and the PSA‐NCAM complex can be used as a predictor of nerve cell repair.^13^ Furthermore, PSA has been reported to regulate NCAM‐positive cells in the liver ducts and then activate hepatic progenitor cells to migrate for regeneration in liver injury.^19^ The liver and salivary glands, as well as the pancreas, originate from the endoderm. Studies have found that endoderm‐derived tissue progenitor cells share similarities in molecular markers and tissue locations.^34^ In the present study, we detected the expression of NCAM and PST under normal conditions. NCAM was mainly expressed on the acinar cells and nerve fibres around the ducts, and PST was indeed expressed in the cytoplasm of salivary duct epithelial cells. The expression of NCAM was increased during the entire regeneration process in the innervation group and was similar to that in the non‐treated glands at day 28 after deligation. Interestingly, PST followed the same tendency as NCAM in the innervation group, and the expression of PST is located in the cytoplasm and nucleus. Jaako K et al indicate that the PST protein nucleus expression was also found in the wild‐type neuroblastoma cells.^35^ However, in the denervation group, the level of NCAM expression decreased during the regeneration process. In contrast, the level of PST expression increased after deligation and remained high at day 28. These data were consistent with the RT‐PCR analysis. Stao C et al revealed that the expression regions and levels of PSA and NCAM are different in different growth stages in the brain.^36^ The expression of PSA and NCAM in salivary glands is still unclear. Our results revealed that the parasympathetic regulation of gland regeneration was associated with increased NCAM and PST. After cutting off the CL, the low expression of NCAM leads to low PSA‐NCAM integration, which decreases the migration of ductal cells and prevents the gland from regenerating. However, STX has been reported to dominate in the embryonic and early postnatal mouse, whereas PST prevails in the adult.^37^ Moreover, the main polysialyltransferases were PST in the damaged livers.^19^ Our research also confirmed that the level of STX expression remained unchanged during gland regeneration. However, the function of altered expression and localization of PST in salivary gland are still unclear. Besides, the specific mechanism by which the parasympathetic nerve promotes gland repair is still unclear. Hence, further studies are needed to explore the mechanism and function of PSA‐NCAM in salivary glands.

In conclusion, we found that the SMG regeneration ability and the proliferation of ductal cells decreased significantly after parasympathetic nerve injury. The proliferation patterns of ductal cells differed obviously between parasympathetic innervation and denervation. Furthermore, the expression of NCAM was reduced after parasympathetic denervation. Our data are the first to confirm the location of NCAM and PST in the salivary glands and show that PSA‐NCAM might one of the mechanisms by which the parasympathetic nerve promotes salivary gland regeneration. Meanwhile, the level of PSA‐NCAM may be a potential indicator of salivary gland regeneration after nerve injury.

## CONFLICT OF INTEREST

The authors declare that they have no conflict of interest.

## AUTHOR CONTRIBUTIONS

XW performed the experiments and wrote the manuscript. ZL and QS aided in the process of experiments and collected the samples. XW, CZ and JW analysed the data and reviewed the manuscript. ZH, SW and LQ designed and supervised the project. All authors approved the final version of the manuscript.

## Supporting information

Fig S1

Fig S2

Fig S3

Fig S4

Table S1

Supplementary Material

## Data Availability

The data that support the findings of this study are available from the corresponding author upon reasonable request.
