# Supplementary figures and images for: The intact parasympathetic nerve promotes submandibular gland regeneration through ductal cell proliferation
Source: Cell Prolif. 2021 Jun 7;54(7):e13078. doi: 10.1111/cpr.13078 (PMC8249781; doi:10.1111/cpr.13078)

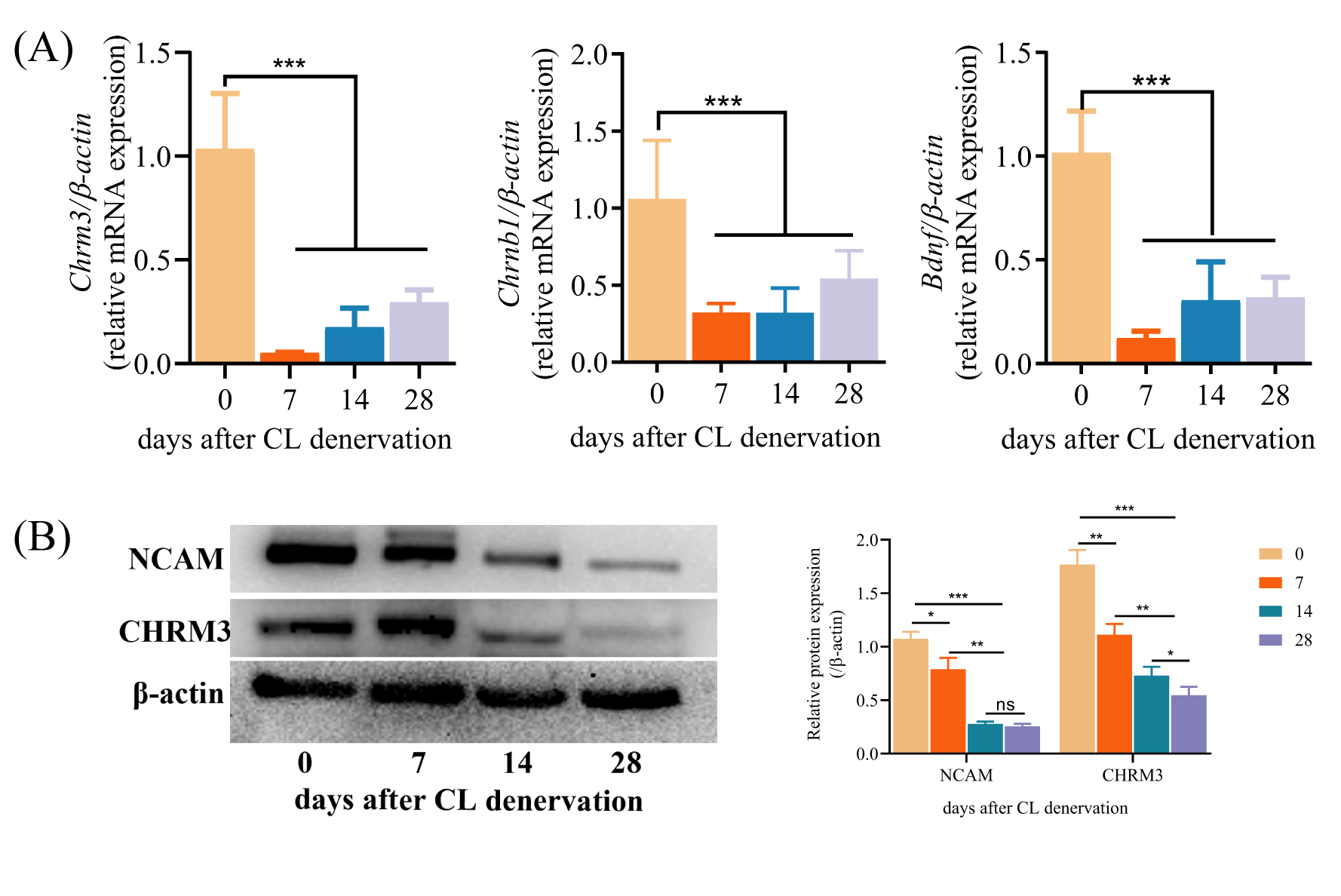

Supplement: Supplementary file 1 — Fig S1 [file CPR-54-e13078-s003.tif]

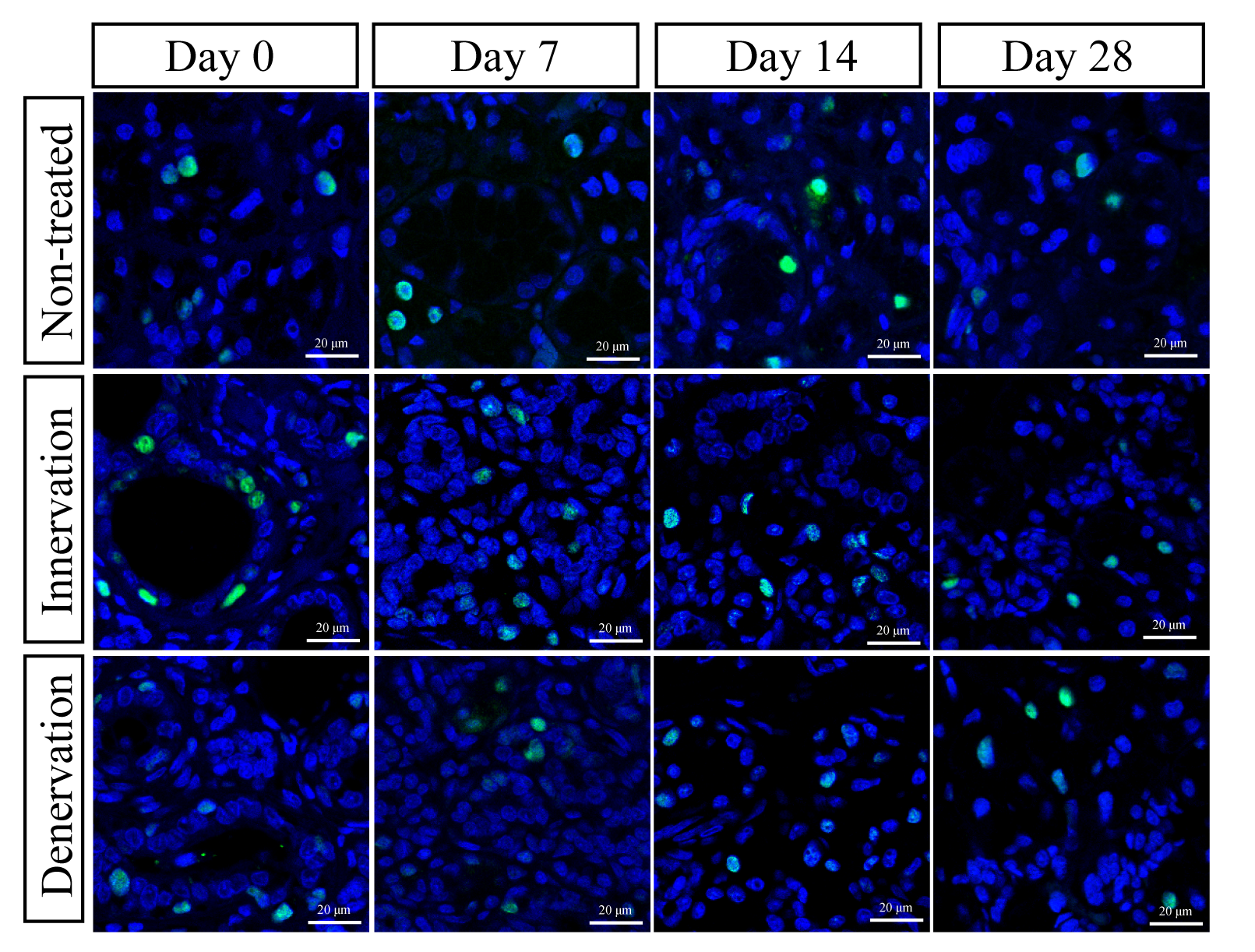

Supplement: Supplementary file 2 — Fig S2 [file CPR-54-e13078-s001.tif]

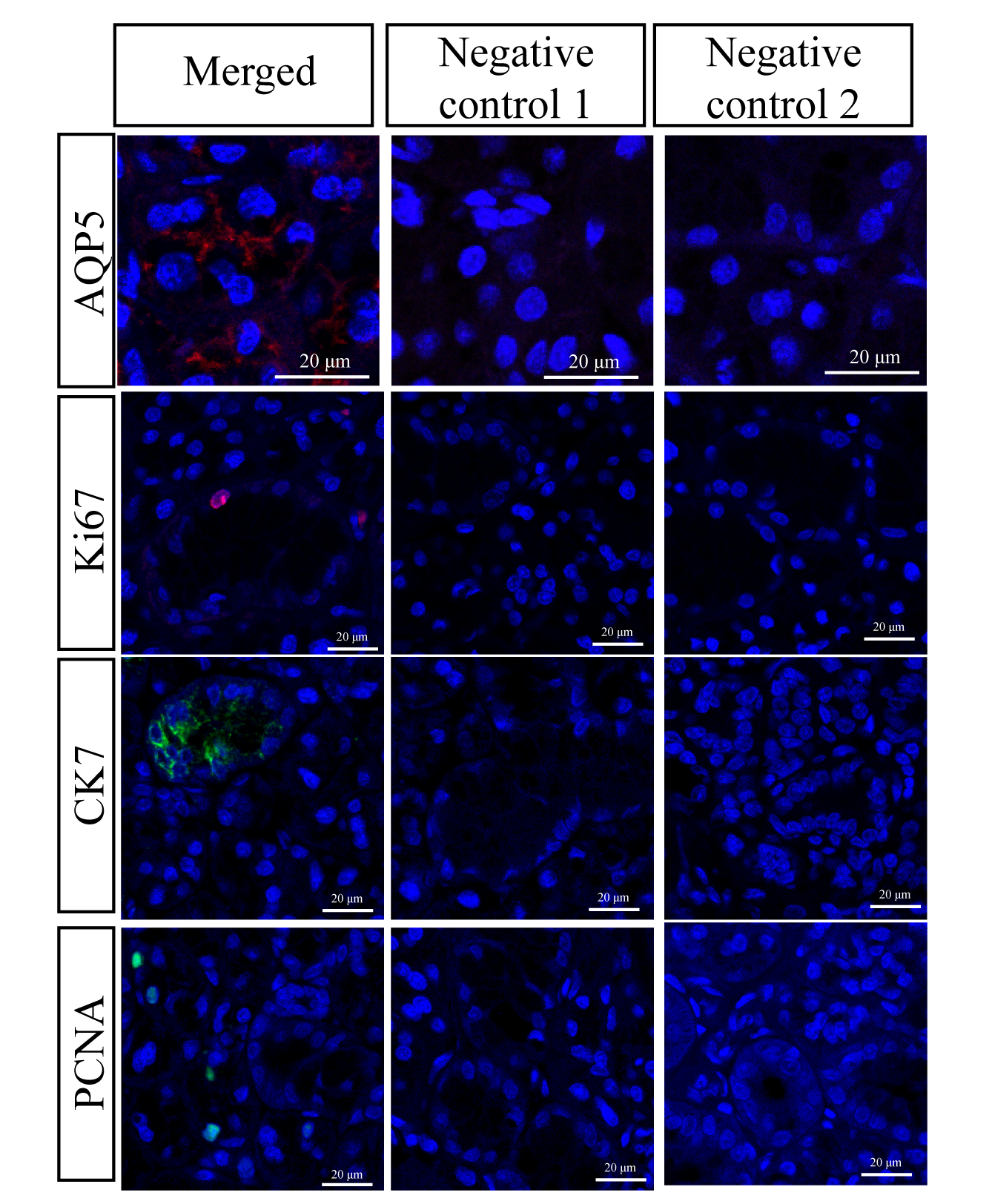

Supplement: Supplementary file 3 — Fig S3 [file CPR-54-e13078-s005.tif]

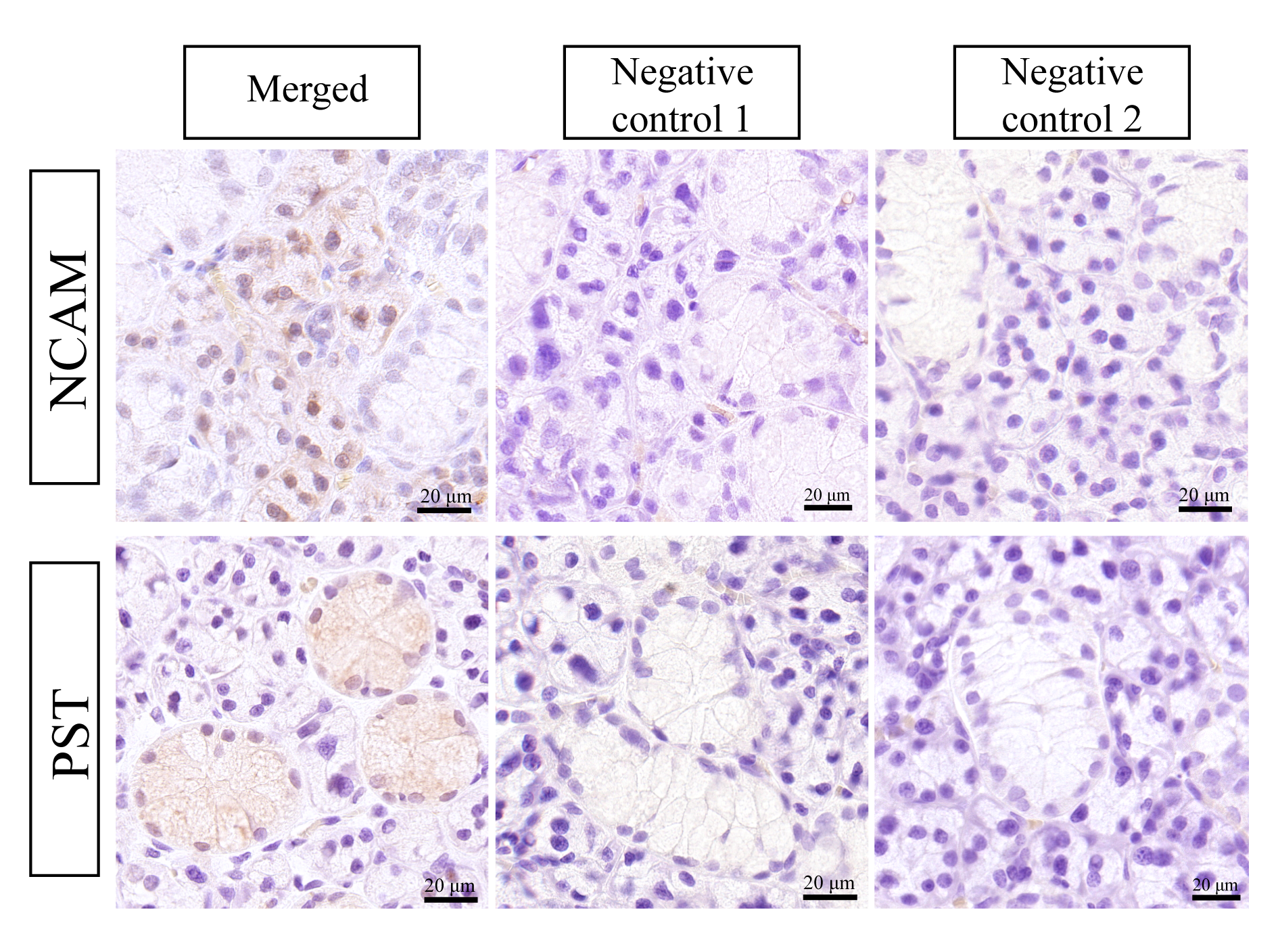

Supplement: Supplementary file 4 — Fig S4 [file CPR-54-e13078-s002.tif]
