# Supplementary material for: The intact parasympathetic nerve promotes submandibular gland regeneration through ductal cell proliferation
Source: Cell Prolif. 2021 Jun 7;54(7):e13078. doi: 10.1111/cpr.13078 (PMC8249781; doi:10.1111/cpr.13078)
Supplement: Supplementary file 5 — Table S1 [file CPR-54-e13078-s006.docx]

**Table S1.** Primers were listed as below:

| Name | F/R | Sequences |
| --- | --- | --- |
| *β-actin* | F | 5′-GTGACGTTGACATCCGTAAAGA-3′ |
|  | R | 5′-GCCGGACTCATCGTACTCC-3′ |
| *Aqp5* | F | 5′-GCCCTCTTAATAGGCAACCAG-3′ |
|  | R | 5′-GCATTGACGGCCAGGTTAC-3′ |
| *Ncam* | F | 5′-AGAAATCAGCGTTGGAGAGTCC-3′ |
|  | R | 5′-TCGTCATCATTCCACACCACT-3′ |
| *Pst* | F | 5′-ATGCGCTCAATTAGAAAACGGT-3′ |
|  | R | 5′-CTCCGATGAGTTGCGTCTCTT-3′ |
| *Stx* | F | 5′-TCGCTGACAGAAGTAATGAAAGC-3′ |
|  | R | 5′-TCAGAGAGAGCGTCTGGTTGT-3′ |
| *Bdnf* | F | 5′-CCCGGTATCAAAAGGCCAAC-3′ |
|  | R | 5′-GTAGTTCGGCATTGCGAGTT-3′ |
| *Chrm3* | F | 5′-GCCTTCATCATCACGTGGAC-3′ |
|  | R | 5′-CATAGCACACAGGGTTCACG-3′ |
| *Chrnb1* | F | 5′-GGACCTACGACCGTACTGAG-3′ |
|  | R | 5′-GAGTACGCAGGGGATGATGA-3′ |
