# Supplementary material for: The intact parasympathetic nerve promotes submandibular gland regeneration through ductal cell proliferation
Source: Cell Prolif. 2021 Jun 7;54(7):e13078. doi: 10.1111/cpr.13078 (PMC8249781; doi:10.1111/cpr.13078)
Supplement: Supplementary file 6 — Supplementary Material [file CPR-54-e13078-s004.docx]

**Appendix Figure S1** The expression of parasympathetic nerve-related genes was decreased after the chorda lingual (CL) denervation. (A) CHRM3, CHRNB1 and BDNF mRNA levels in different times after CL injury without duct ligation/deligation. CL, chorda lingual. (B) Western blot detected the relative protein expression of CHRM3 and NCAM in the glands. *** indicates significance at P < 0.001, ** P<0.01, * P<0.05, ns for no significant. Data were shown as mean ± standard deviation.

**Appendix Figure S2** The PCNA expression in the regenerating glands. Immunofluorescence (IF) of proliferating cell nuclear antigen (PCNA) and DAPI (blue) in the gland of nontreated, innervation and denervation group at 0, 7, 14, and 28 days after deligation. Scale bar = 20 μm.

**Appendix Figure S3** The negative control of AQP5, Ki67, CK7 and PCNA. For negative control 1, we used PBS instead of the primary antibody was used as the secondary antibody only control. Mouse monoclonal anti-IgG instead of the primary antibody was used as negative control 2. Scale bar = 20 μm.

**Appendix Figure S4** The negative control of NCAM and PST. For negative control 1, we used PBS instead of the primary antibody was used as the secondary antibody only control. Mouse monoclonal anti-IgG instead of the primary antibody was used as negative control 2. Scale bar = 20 μm.
